# Longitudinal study on the change trend of serum alkaline phosphatase and its possible influencing factors in peritoneal dialysis patients

**DOI:** 10.1038/s41598-024-63721-5

**Published:** 2024-06-07

**Authors:** Wei Zhao, Sen Zhang, Hai-dan Zhao

**Affiliations:** Department of Nephrology, Peking University Shougang Hospital, Shijingshan District, Beijing, 100144 China

**Keywords:** Peritoneal dialysis, Repeated measurements, Serum alkaline phosphatase, Kidney, Renal replacement therapy, Peritoneal dialysis, Nephrology

## Abstract

The aim of the study was to analyze the change trend of serum ALP over time and identify factors influencing its levels in peritoneal dialysis patients. Then to investigate the impact of serum ALP changes on calcium and phosphorus metabolism in single peritoneal dialysis center utilizing repeated measurement data. A retrospective cohort study was conducted with a total follow-up duration of 30 months. Serum ALP and other biomarkers, including calcium (Ca), phosphorus (P), 25(OH)D, intact parathyroid hormone (iPTH), albumin(ALB), and hemoglobin(Hb) were measured every 3 months. The generalized estimation equation (GEE) was utilized to analyze the change trend of serum ALP over time, and to assess whether there were differences in changes over time between different genders and different primary disease groups. Additionally, factors influencing serum ALP levels were analyzed, and the impact of serum ALP changes on calcium and phosphorus metabolism was also explored. A total of 34 patients were included in the study. Serum ALP and other indicators were measured repeatedly, with a maximum of 8 times and a minimum of 4 times. The median of serum ALP values at all measurement times for all selected patients was 89 U/L. The GEE analysis revealed that serum ALP gradually increased with time, and patients in diabetes group increased faster than those in non-diabetes group. A positive correlation was observed between serum ALP and dialysis duration, also between serum ALP and hemoglobin. However, variations in serum ALP did not significantly affect serum corrected calcium, phosphorus, or iPTH concentrations. The serum ALP levels of peritoneal dialysis patients increase gradually over time, and the concentrations are influenced by dialysis duration. The changes in serum ALP values do not have a significant impact on serum calcium, phosphorus, and iPTH levels.

## Introduction

Peritoneal dialysis, as an important way of renal replacement therapy, has got increasing attention due to its flexibility. Peritoneal dialysis patients commonly combine with abnormal mineral and bone metabolism (CKD-MBD), which affects their life quality and long-term prognosis. The intricate mechanisms underlying CKD-MBD remain incompletely understood and continue to be a pivotal area of research. Alkaline phosphatase (ALP) as an indicator of CKD-MBD has received more attention in recent years^1^. Emerging studies have found that ALP not only participates in bone and cartilage calcification, but also in calcification of soft tissues such as blood vessels. Moreover, ALP is intricately linked to inflammation, metabolic syndrome, and other pathophysiological processes^2,3^. Notably, serum ALP levels serve as a crucial prognostic indicator, predicting the development and progression of adverse clinical outcomes in peritoneal dialysis patients^4^. And Peritoneal dialysis patients usually have abnormal serum ALP levels, which is associated with an increased risk of all-cause mortality^5–7^. So we must attach importance to serum ALP and conduct research into when and why the concentrations become abnormal. This constitutes the purpose of our study. In this study, we utilized repeated measurement data to examine the change trends in serum ALP levels among peritoneal dialysis patients, the factors influencing serum ALP levels, and the impact of serum ALP changes on calcium and phosphorus metabolism.

## Materials and methods

### Subjects

We selected stable peritoneal dialysis patients from Peking University Shougang Hospital, who can be followed up regularly from January 2020 to July 2022. Inclusion criteria: ① Age >  = 18 years old, <  = 80 years old; ② at least one year of consistent follow-up; ③ during the entire follow-up period, no liver and gallbladder diseases or fractures occurred. Exclusion criteria: Patients with chronic wasting diseases such as tumors, tuberculosis, or chronic liver disease, or those unable to adhere to regular follow-ups. At enrollment, we collected general patient data, including age, gender, dialysis vintage, and whether the primary disease was diabetic nephropathy or not. All selected patients were treated with Bette's lactate peritoneal dialysis solution, utilizing glucose as an osmotic agent in either 1.5% or 2.5% concentrations. Each patient underwent 4–5 exchanges per day, with 2000 mL per exchange. The dialysis solution comprised 135 mmol/L sodium, 1.25 mmol/L calcium, 0.5 mmol/L magnesium, and 35 mmol/L lactate.

### Follow-up and biomarkers

The total follow-up period was 30 months. We conducted a face-to-face interview for every subject each 3 months which derived from our routine management of PD patients cohort. Patients were required to visit at least four times consecutively. Failure to meet this requirement led to exclusion. During each visit, patients were inquired about recent occurrences of fractures, liver and gallbladder diseases, acute infections, or acute cardiovascular events. If any of these conditions were reported, an additional visit was scheduled within one month, following resolution of the condition. Fasting blood samples were collected during each visit, and all indicators of mineral and bone metabolism were tested. These indicators encompassed serum alkaline phosphatase (ALP), 25(OH)D, intact parathyroid hormone (iPTH), corrected blood calcium (Ca), blood phosphorus (P), and serum creatinine (Cr). Within an hour of sample collection, the specimen is promptly dispatched to the hospital’s laboratory for testing. The automatic analyzer is utilized to measure serum levels of calcium (Ca), phosphorus (P), alkaline phosphatase (ALP), and creatinine (Cr). Specifically, serum ALP is determined using a colorimetric method. Additionally, the Roche fully automated electrochemiluminescence immunoassay analyzer is employed to quantify serum 25(OH)D levels, while serum intact parathyroid hormone (iPTH) is assessed through a chemiluminescence assay. To calculate corrected calcium levels, the formula Correction calcium (mmol/L) = measured calcium−0.02 × [ALB (g/L)−40] is applied.

### Statistics analyses

For statistical analyses, measurement data were presented as mean ± standard deviation, while categorical variables were reported as frequencies and percentages. To analyze the change trends of serum ALP over time, the generalized estimation equation (GEE) was employed, taking into account the number of repeated measurements as a categorical variable. Relevant variables were respectively added to the model, and different models were performed to examine whether there were differences in change trend over time between different genders and between different primary diseases.The study further explored factors influencing serum ALP levels and assessed the impact of serum ALP changes on calcium and phosphorus metabolism. When using GEE, selected the appropriate work-related structure based on the minimum QIC value. All statistical computations are carried out using SPSS 25.0 software.

## Results

### Baseline patient characteristics

For this study total 34 patients met inclusion and follow-up criteria, including 9 females and 25 males, ranging in age from 42 to 80 years, with mean and median age both of 58 years. None of the participants had undergone hemodialysis or kidney transplantation prior to the study. During the 30-month follow-up period, 23 patients initiated peritoneal dialysis and completed at least one year of follow-up. The least dialysis vintage of subjects was 12 months and the maximum was 122 months, with a median of 26 months and a three-quarter duration of 42 months. The average dialysis duration was 34 months. Of the 34 patients, 24 suffered from diabetic nephropathy as the primary disease, and the other 10 patients were hypertensive kidney damage and chronic nephritis. The peritoneal dialysis Kt/V, residual kidney Kt/V, and average blood biochemical results during the entire follow-up period of these 34 patients were shown in Table 1. Among them, serum ALP levels exhibited a positive skew distribution, with a median of 89 U/L, ranging from the first quartile of 69 U/L to the third quartile of 111 U/L.

**Table 1:** Baseline characteristics of included patients (n = 34) and total mean value of their biochemical indicators in follow-up.

Demographic and clinical characteristics	
Age, years (median)	58 (38, 80)
Sex, men/women	25/9
Diabetes, yes/no	24/10
Incident PD/maintain PD	23/11
Time, n, 1/2/3/4/5/6/7/8	34/34/34/34/29/23/17/14
PD Kt/v	1.18 ± 0.38
RF Kt/V	0.76 ± 0.82
Total Kt/V	1.94 ± 0.88
Biochemical indicators in follow-up	
ALP (U/L) (n = 219)	108 ± 78
Cr (umol/L) (n = 219)	899 ± 266
25(OH)D (ng/ml) (n = 219)	7.45 ± 4.43
ALB (g/L) (n = 219)	36.0 ± 4.24
Hb (g/L) (n = 219)	115 ± 20
CRP (mg/L) (n = 219)	3.41 ± 2.82
Adjusted Ca (mmol/L) (n = 219)	2.18 ± 0.21
P (mmol/L) (n = 219)	1.76 ± 0.46
iPTH (ng/ml) (n = 219)	175.91 ± 131.64

### Follow-up profile

During the observation period of 30 months, all patients underwent regular testing for mineral and bone metabolism indicators every 3 months, and the drug type and dose of phosphorus binders were adjusted based on serum calcium and phosphorus levels. Furthermore, the adequacy of peritoneal dialysis was assessed for each patient every six months, and any necessary improvements to the dialysis protocol were implemented base on the assessment. Notably, the overall serum iPTH value of the included patients was low throughout the follow-up period. Except for 3 patients who used calcium mimetics, the remaining patients did not utilize decreasing iPTH drugs, and all selected patients did not take active vitamin D or its analogues. The most follow-up was 8 times, with a total of 14 people finished. The least follow-up was 4 times, and all 34 people completed more than 4 follow-up visits.

### Serum ALP changes trend, affecting factors and its impact on CKD-MBD

The change trend of serum ALP in peritoneal dialysis patients over time was shown in Table 2. Our findings indicated that serum ALP values increased with peritoneal dialysis duration extends across all study populations, but the trend was non-linear. Specifically, the rate of increase slowed down after the initial year, as evidenced by Model 1 in Table 2. Grouping subjects by gender, i.e. in Model 2, the change trend of serum ALP between males and females showed no significant difference over time. Divided them into two teams according to whether they were combined with diabetes or not, as shown in Model 3, serum ALP in the diabetes nephropathy group increased faster than that in the non-diabetes group, and the difference was statistically significant.

**Table 2:** Analysis of serum ALP change trend over time by GEE.

Variable	Model1^a^ B (95%CI)	Wald Chi-Square	*p*	Model2^b^ B (95%CI)	Wald Chi-Square	*p*	Model3^c^ B (95%CI)	Wald Chi-Square	*p*
Time	/	5.171	0.023*	2.702 (0.032 to 5.37)	3.934	0.047*	− 0.842 (− 2.66 to 0.97)	0.826	0.363
Sex (reference, male)				− 2.07 (− 43.4 to 39.3)	0.010	0.922			
Sex*time				0.227 (− 4.56 to 5.02)	0.009	0.926			
Diabetes or not (reference, no Diabetes)							− 13.54 (− 52.2 to 25.1)	0.472	0.492
Diabetes*time							4.963 (1.04 to 8.88)	6.152	0.013*

Working correlation matrix: AR(1). *: *p* < 0.05.

^a^Model 1: intercept, time. TIME as factors and Contrast Polynomial is Quadratic.

^b^Model2: intercept, time, sex, sex*time. TIME as Covariates.

^c^Model3: intercept, time, diabetes or not, diabetes*time. TIME as Covariates.

The analysis of factors influencing serum ALP in peritoneal dialysis patients was presented in Table 3. In this model, we incorporated factors that may theoretically affect ALP levels into the equation and conducted a regression analysis. Adhering to the GEE equation, the smaller the QIC value, the better the model. Right work-related structure and meaningful covariates were selected. The results ultimately revealed that dialysis vintage and hemoglobin were independent factors influencing serum ALP levels in peritoneal dialysis patients. Specifically, dialysis vintage was positively correlated with serum ALP levels, and with the dialysis duration prolonging, serum ALP levels increasing. Similarly, hemoglobin was positively associated with serum ALP, meaning that an elevation in hemoglobin was accompanied by an increase in serum ALP. However, corrected serum calcium, serum phosphorus, and iPTH levels did not exert any significant effect on serum ALP values.

**Table 3:** Analysis of factors affecting serum ALP by GEE.

Variable^a^	B (95%CI)	Wald Chi-Square	*p*
Diabetes			
Yes	31.39 (− 8.533, 71.31)	2.375	0.123
No	Reference		
Sex			
Male	12.48 (− 50.21, 75.17)	0.152	0.696
Female	Reference		
Age (years)	1.107 (− 0.558, 2.773)	1.698	0.193
PD vintage (months)	1.223 (0.314, 2.132)	6.950	0.008^b^
Time	2.929 (0.051, 5.806)	3.980	0.046^b^
P Kt/V	− 27.29 (− 109.9, 55.33)	0.419	0.517
RF kt/V	− 9.540 (− 32.22, 13.14)	0.679	0.410
Cr( mmol/L)	− 0.015 (− 0.044, 0.013)	1.069	0.301
Hb (g/L)	0.319 (0.041, 0.596)	5.064	0.024^b^
ALB (g/L)	0.935 (− 0.619, 2.036)	1.391	0.238
CRP (mg/L)	0.368 (− 1.301, 0.003)	0.186	0.666
25(OH)D (ng/ml)	0.266 (− 1.033, 1.566)	0.161	0.688
P (mmol/L)	− 0.808 (− 11.74, 10.12)	0.021	0.885
Adjusted Ca (mmol/L)	− 0.018 (− 2.610, 2.574)	0.000	0.989
iPTH (ng/mL)	0.023 (− 0.009, 0.056)	1.931	0.165

Working correlation matrix: AR(1).

^a^Separately enter adjusted Ca, P, iPTH into the model, the model was not improved and the regression coefficients of these variables were not significant.

^b^*p* < 0.05.

The impact of serum ALP on calcium and phosphorus metabolism was shown in Table 4. In Model 1, where corrected calcium served as the dependent variable, factors that may affect serum corrected calcium were incorporated to GEE multiple regression analysis, including serum ALP levels. The findings revealed that serum ALP levels in peritoneal dialysis patients did not significantly affect serum corrected calcium. In Model 2 with serum phosphorus as the dependent variable, ALP entered the regression equation as the independent variable, and the results showed that its value change had no significant impact on serum phosphorus. Similarly, in Model 3, focusing on blood iPTH as the dependent variable, the factors affecting serum iPTH levels were analyzed, and the results showed that changes in serum ALP did not significantly influence serum iPTH levels.

**Table 4:** Analysis of the effect of serum ALP on serum adjusted calcium, phosphorus, and iPTH by GEE.

	Model1^a^ dependent variable adjusted Ca			Model2^b^ dependent variable P			Model3^c^ dependent variable iPTH		
	B (95%CI)	Wald Chi-Square	*p*	B (95%CI)	Wald Chi-Square	*p*	B (95%CI)	Wald Chi-Square	*p*
Time (as factors)	–	2.088	0.955	–	9.937	0.192	–	7.122	0.416
ALP (U/L)	0.000 (− 0.003, 0.002)	0.220	0.639	0.000 (− 0.001, 0.000)	0.497	0.481	0.070 (− 0.067, 0.207)	1.012	0.314

Working correlation matrix: AR(1).

^a^Model 1 adjusted for sex, diabetes or not, age, PD vintage, P, Total Kt/V, ALB, CRP,25(OH)D.

^b^Model 2 adjusted for sex, diabetes or not, age, PD vintage, adjusted Ca, Total Kt/V, iPTH, ALB, CRP, 25(OH)D.

^c^Model 3 adjusted for sex, diabetes or not, age, PD vintage, adjusted Ca, P, Total Kt/V, ALB, CRP, 25(OH)D.

## Discussion

As is well known, four ALP isozymes are expressed in humans: tissue-nonspecific ALP, intestinal ALP(IALP), placental ALP and germ cell ALP. Serum ALP mainly includes bone specific ALP and hepatogenic ALP, with an activity ratio of approximately 1:1, comprising more than 90% of the total. The remaining circulating ALP activity, 1–10%, is attributed mostly to IALP, which appear frequently in dialysis patients. It should be kept in mind for nephrologists and with the increases in the detectability of the intestinal type, we can explore serum ALP more accurately. Osteogenic ALP (BALP) is a glycoprotein secreted by osteoblasts. The serum BALP level can reflect the activity of osteoblasts, and its main physiological function is to promote the occurrence of calcification in the body, including bone and cartilage calcification, as well as soft tissues calcification such as blood vessels, tendons, and ligaments. Elevated serum ALP caused by hepatogenic diseases is often accompanied by other liver enzyme abnormalities. In the absence of liver disease, the increase in serum total ALP levels is mainly caused by an increase in BALP. In this situation serum total ALP can be used as a prognostic indicator for a range of illnesses. In the last two decades, many studies have revealed that serum total ALP level are associated with cardiovascular events and all-cause mortality, whether in the general population, non-dialysis CKD population, hemodialysis population, or peritoneal dialysis population^8–11^. Therefore, researchers pay more attention to serum total ALP and even explored it as a target for drug therapy recently^12^. In addition, in CKD patients, serum ALP raising also indicates renal osteodystrophy occurrence, and can be used to diagnose the type of renal bone disease, especially combined with serum iPTH, to predict the risk of fracture and mortality. So nephrology doctors pay more and more attention to serum ALP levels and make a large number of researches about it^13,14^. But most of these studies were based on cross-sectional serum ALP values, and it is unclear whether serum ALP changes gradually over time or not and what the pattern of change trend is. Our study investigated serum ALP change trend over time by utilizing repeated measurement data and the influencing factors of serum ALP in peritoneal dialysis patients.

This study observed the change trend of serum ALP over time, and the results indicated that during the 30-month follow-up period, the serum ALP values measured every 3 months gradually increased with time, but were non-linear, and the increase rate decelerated in the later stage. The variations of serum ALP over time was statistically significant. This suggested that if someone want to study the clinical significance of serum ALP in peritoneal dialysis patients, especially cross-sectional studies or prognostic studies that rely on cross-sectional values, Attention needed to be paid to the dialysis stage where the measurement point was located. Previous studies have showed that serum ALP rose with increasing dialysis vintage^15^. So we thought the increase of serum ALP values over time may be partially attributed to the accumulation of dialysis vintage, that is, as dialysis duration prolongs, CKD-MBD gradually progresses and soft tissue calcification also occurs. In addition, because more than two-thirds of the selected patients in our study were newly enrolled in peritoneal dialysis, the change trend also reflects the development trend of CKD-MBD, which was faster in the early stage and slower in the later stage. After adjusting for the variable of time, we found no significant difference in serum ALP levels between male and female patients, and the change trend over time between them had no difference. This finding was inconsistent with previous research conducted in general populations which revealed that serum ALP levels increase more rapidly among women over 40 years old^16^. However, in patients with diabetes nephropathy as the primary disease, serum ALP concentration increased faster over time than those without diabetes nephropathy. And there were no significant differences in terms of age, gender ratio, dialysis duration, or dialysis adequacy between the diabetes nephropathy group and the non-diabetes nephropathy group (shown in supplement 1). It is plausible that patients with diabetes nephropathy are more prone to CKD-MBD, particularly ectopic calcification, which is linked to the micro-inflammatory state and insulin resistance common in diabetic patients. Recent investigations have also demonstrated that serum ALP levels are associated with the development of diabetes in the general population, and high ALP levels serve as independent risk factors for poor prognosis in diabetes nephropathy^17^. Therefore, serum ALP should be closely monitored in diabetic patients undergoing peritoneal dialysis.

Then we explored the factors that may have impact on serum ALP levels utilizing longitudinal measurement data and GEE analysis. Our findings revealed that dialysis vintage emerged as an independent risk factor for an increase in serum ALP levels. As the duration of dialysis prolonged, the concentration of serum ALP rose, which was consistent with previous studies^18^. Other factors that may affect serum ALP level, such as age, gender, total Kt/V, creatinine, albumin, etc., were also included in the equation, and the results shown that a positive correlation between blood hemoglobin and serum ALP. Previous studies have hinted that higher serum ALP level may indicate better nutritional state among non-CKD patients with higher hemoglobin levels^19^. Another study observed a positive correlation between serum ALP and serum albumin among peritoneal dialysis patients with residual kidney function^20^. These findings suggested that serum ALP levels could serve as an indicator of nutritional status in specific peritoneal dialysis populations at particular time points. Notably, two-thirds of our study participants were newly initiated on peritoneal dialysis, suggesting that serum ALP levels in this subgroup may be linked to nutritional status. Specifically, individuals with higher ALP levels exhibited higher hemoglobin levels. However, our study also indicated that serum corrected calcium, blood phosphorus, iPTH, and blood 25 (OH) D all were not predictive factors for serum ALP levels in peritoneal dialysis patients. Researchers had made great effort to clarify the association between the above indicators in previous cross-sectional and prognostic studies, but the results were controversial. Some studies found that the group with elevated serum ALP had lower levels of serum phosphorus and calcium^4,6^, while others found that the group with higher serum ALP has higher levels of blood calcium^21^ or a higher incidence of hyperphosphatemia^15^. These inconsistent findings may attribute to the varying serum calcium and phosphorus levels and diverse therapeutic drugs in different clinical studies. Past most studies suggested a positive correlation between serum ALP and iPTH^4,6,15,18^, while others had the opposite conclusions^21^. In our study, we did not find that serum iPTH levels had impact on serum ALP concentrations, which may be attributed to the fact that most patients in our cohort were newly initiated on dialysis, typically having low serum iPTH levels, and only a minority received calcium mimetic agents to reduce iPTH. In addition, the study revealed that serum 25 (OH) D was not a predictive factor for serum ALP levels. In this study, serum 25 (OH) concentrations were generally low and did not exhibit significant temporal variations. Several researches have similarly found that the serum 25 (OH) D levels were low in the high ALP group, which may be due to inflammatory status^19^.In summary, the interplay between CKD-MBD indicators is complex and the studies findings about the relationship were inconsistent due to different dialysis stages and clinical interventions. Our study primarily focused on newly entered peritoneal dialysis patients, with diabetes nephropathy as the primary diagnosis. Their serum calcium and phosphorus levels were mostly managed to recommended ranges, serum iPTH and 25(OH)D concentrations were predominantly low, and most total Kt/V were up to recommended standards. On above conditions, the serum ALP values increased over the dialysis duration, though the trend was not linear, being more rapid in the early stage and slower in late stage during the 30-month follow-up. Dialysis duration emerged as an independent influencing factor, positively correlated with serum ALP.

Next, we investigated the impact of serum ALP on calcium and phosphorus metabolism. Our findings revealed that in clinical real world where drugs and treatment were often utilized to maintain serum calcium, phosphorus and the whole parathyroid gland levels up to recommended values, after accounting for other confounding factors, the serum ALP level exhibited no significant influence on the serum adjusted calcium, phosphorus and iPTH concentrations. This underscored the intricate nature of mineral and bone metabolism abnormalities in peritoneal dialysis patients, along with the independence of serum ALP as a biomarker. Serum ALP levels not only reflect calcium and phosphorus metabolism, but also nutrition condition, inflammation status, and cardiovascular complications.

The novelty of this study lies in utilizing repeated measurement data derived from long-term follow-up in peritoneal dialysis patients to examine the change patterns of serum ALP over time and the factors influencing its levels, which have been scarcely in previous research. The clinical significance of the study lies in the longitudinal observation of serum ALP in peritoneal dialysis patients, producing more comprehensive characteristics of this biomarker and serving as a reference for future therapeutic interventions. However, it is noteworthy that the study was limited by the relatively small sample size and potential heterogeneity among participants, comprising both newly initiated and long-term maintenance dialysis patients. As such, more subjects and a stratified design are needed for further research.

## Conclusions

The serum ALP levels in peritoneal dialysis patients gradually increased over time, and the rise was faster in the population whose primary disease were diabetes nephropathy. Dialysis vintage is an independent factor affecting serum ALP concentration, and it is positively correlated with serum ALP. Longitudinal data analysis showed that changes in serum ALP had no significant impact on serum adjusted calcium, blood phosphorus, and serum iPTH values.

### Supplementary Information

Supplementary Table 1.
