# Supplementary material for: Longitudinal study on the change trend of serum alkaline phosphatase and its possible influencing factors in peritoneal dialysis patients
Source: Sci Rep. 2024 Jun 7;14:13099. doi: 10.1038/s41598-024-63721-5 (PMC11161618; doi:10.1038/s41598-024-63721-5)
Supplement: Supplementary file 1 — Supplementary Table 1. [file 41598_2024_63721_MOESM1_ESM.docx]

**Supplement 1 Comparison of general data between diabetic patients and non-diabetic patients**

|  | diabetic patients | non-diabetic patients | test | *p* |
| --- | --- | --- | --- | --- |
| Age, years（median） | 58 | 61 | *t*=0.673 | 0.505 |
| Sex, male/female | 18/6 | 7/3 | Fisher’s test | 0.538 |
| Dialysis duration,months | 27 | 34 | Mann-Whitney U test | 0.137 |
| Total Kt/V | 1.97±0.97 | 1.89±0.65 | *t*=-0.243 | 0.809 |
